# San Wu Huangqin Decoction, a Chinese Herbal Formula, Inhibits Influenza a/PR/8/34 (H1N1) Virus Infection In Vitro and In Vivo

**DOI:** 10.3390/v10030117

**Published:** 2018-03-09

**Authors:** Qinhai Ma, Qingtian Yu, Xuefeng Xing, Sinian Liu, Chunyu Shi, Jiabo Luo

**Affiliations:** 1 School of Traditional Chinese Medical Science, Southern Medical University, Guangzhou 510515, China; 13268268214@163.com (Q.M.); dkdklm@163.com (Q.Y.); xiaoxing0610@163.com (X.X.); scy0169@163.com (C.S.); 2 Guangdong Provincial Key Laboratory of Chinese Medicine Pharmaceutics, Southern Medical University, Guangzhou 510515, China; 3 Biosafety Level-3 Laboratory, School of Public Health, Southern Medical University, Guangzhou 510515, China; liusinian123@163.com

**Keywords:** viral protein, influenza virus, San Wu Huangqin Decoction, viral load

## Abstract

The San Wu Huangqin Decoction (SWHD), a traditional Chinese medicine formula, is used to treat colds caused by exposure to wind-pathogen, hyperpyrexia, infectious diseases and cancer; moreover, it is used for detoxification. The individual herbs of SWHD, such as *Sophora flavescens* and *Scutellaria baicalensis*, exhibit a wide spectrum of antiviral, anti-inflammatory, antibacterial, anticancer and other properties. The Chinese compound formula of SWHD is composed of *S. flavescens, S. baicalensis* and *Rehmannia glutinosa*. However, the effect of SWHD on the influenza virus (IFV) and its mechanism remain unknown. The aim of this study was to evaluate, for the first time, whether SWHD could be used to treat influenza. Results showed that SWHD could effectively inhibit influenza A/PR/8/34 (H1N1) virus at different stages of viral replication (confirmed through antiviral effect assay, penetration assay, attachment assay and internalization assay) in vitro. It could reduce the infection of the virus in a dose- and time-dependent manner, as confirmed by observing the cell cytopathic effect and calculating the cell viability (*p* < 0.05). SWHD demonstrated better antiviral activity than oseltamivir in the evaluation of antiviral prophylaxis on influenza (*p* < 0.05). The antiviral activity of SWHD may be related to its regulation ability on the immune system. Western blot, real-time polymerase chain reaction and indirect immunofluorescence assay showed that the expression of the four target viral proteins of the IFV (namely, haemagglutinin (HA), neuraminidase (NA), nucleoprotein (NP) and matrix-2 (M2)) reduced significantly (*p* < 0.05). Moreover, SWHD (23.40 and 11.70 g/kg) significantly alleviated the clinical signs, reduced the mortality and increased the survival time of infected mice (*p* < 0.05). The lung index, virus titres, pathological changes in lung tissues and the expression of key proteins of the IFV in mice also decreased (*p* < 0.05). In conclusion, SWHD possessed anti-influenza activity. This work provided a new view of complementary therapy and drug discovery for clinical treatment.

## 1. Introduction

The influenza virus (IFV) is one of the most common respiratory viruses and causes a high rate of morbidity and mortality. It spreads rapidly and is easily transmitted from person to person, and even through cross-species transmission. IFV can cause many respiratory tract diseases with severe complications during seasonal epidemics every year worldwide and causes millions of deaths. Many antiviral drugs, including neuraminidase (NA) inhibitors and matrix-2 (M2) channel blockers, and vaccinations have been developed to prevent and treat IFV infections [1,2]. However, the restriction on its use (e.g., rate of virus resistance, high cost and drug toxicity) leads to a negative control of the spread of the viruses [3]. Therefore, safe and effective antiviral drugs for the treatment of IFV infections are needed.

Nowadays, natural drugs serve as important alternative therapies in the healing and treatment of various diseases in the last few decades. A large number of reports have shown the significant advantage of developing novel bioactive chemicals that are extracted from natural drugs. Traditional Chinese medicine (TCM) plays an important role in the treatment of disease in China. The use of TCM dates to many years ago and is safe and effective [4,5]. Some extracts of TCM herbs or their isolated constituents have shown significant therapeutic and preventive effects, including antiviral, anti-inflammatory, antibacterial, anticancer and immuno-modulatory properties [6,7]. Therefore, TCM is a promising anti-viral potential against a wide range of viruses, such as H1N1, H5N1 and Sendai virus etc. [8,9]. The San Wu Huangqin Decoction (SWHD), a classic formula for the treatment of cold caused by exposure to wind-pathogen with hyperpyrexia and deficiency of Yin-blood, was first prescribed by Sun Simiao in “Beiji Qianjin Yao Fang”, with a prescription name of “Ku Shen Decoction”. It was then recommended by Zhang Zhongjing in “Jinkui Yaolue”. SWHD is a herbal remedy for replenishing Yin and removing heat and toxic materials. It is composed of *Sophora flavescens*, *Scutellaria baicalensis* and *Rehmannia glutinosa*. Actually, SWHD was initially used for colds and fever in hemopenia puerpera, and its contemporary clinical application has been expanded to infectious diseases, autoimmune diseases, bacterial infectious diseases, inflammation symptoms and cancer. *S. flavescens* and *S. baicalensis*, the main herbal components of SWHD, exhibit a wide spectrum of effects to viruses, such as H1N1, RSV (Respiratory Syncytial Virus), Coxsackie virus B3 and EV71 (Enterovirus 71), due to their major bioactive ingredients, including quinolizidine alkaloids, flavonoids, and triterpenoids [10,11,12,13,14]. The specific combination of different medical substances is the basic theory behind the formulation of TCM treatments. Although the anti-IFV effects of the bioactive ingredients of SWHD have been reported for many years in TCM clinical case studies, the empirical antiviral potential of SWHD should be considered and merits more experimental assessment.

In the present study, a comprehensive evaluation of the anti-influenza A virus activity of SWHD was performed in vitro and in a mouse model of influenza infection. We demonstrated that SWHD inhibited the influenza A virus replication in a concentration- and time-dependent manner by observing the cell cytopathic effect and calculating the cell viability. We further found that the observed anti-influenza activity of SWHD might be correlated with its inhibitory effects on major virus proteins by using real-time polymerase chain reaction (RT-PCR), indirect immunofluorescence, and Western blotting. Moreover, oral administration of SWHD to mice significantly mitigated influenza A virus-induced pneumonia and reduced the viral titres in the lungs. These findings may pave the way for the use of SWHD as a therapeutically effective antiviral to combat influenza A virus infection.

## 2. Materials and Methods

### 2.1. Reagents

Acteoside (94.1%, lot#: 111810-201405), baicalin (93.3%, lot#: 110715-201318), wogonoside (96.2%, lot#: 110753-201415), baicalein (93.3%, lot#: 110715-201318), matrine (96.2%, lot#: 110753-201415), sophocarpine (93.3%, lot#: 110715-201318), oxymatrine (96.2%, lot#: 110753-201415) and oxysophocarpine (93.3%, lot#: 110715-201318) were provided by the National Institutes for Food and Drug Control (Beijing, China). Oseltamivir (lot: 10141721) was provided by F.Hoffmann-La Roche Ltd., Basel, Switzerland and repacked by Shanghai Roche Pharmaceuticals Ltd. (Roche, Switzerland). *Sophora flavescens* (lot#: 170313501), *Scutellaria baicalensis* (lot#: 170404981) and *Radix Rehmanniae* (lot#: 170500931) were supplied by Guangzhou Zhixin Pharmaceutical Co., Ltd. (Guangzhou, China) and authenticated by Professor Ji Ma (Southern Medical University, Guangzhou, China).

### 2.2. Preparation and High-Performance Liquid Chromatography (HPLC) Analysis of SWHD Extract

#### 2.2.1. Sample Preparation for HPLC Analysis

According to the extraction method described in the Beiji Qianjin Yao Fang, *S. flavescens* (6 g), *S. baicalensis* (6 g), and *Rehmannia glutinosa* (12 g) (Guangzhou, China) were decocted twice in 10 volumes of distilled water (*v*/*w*), respectively, and the decoctions were mixed, extracted with 75% ethanol and centrifuged. Then the extract was filtered and freeze-dried using a lyophiliser (Labconco, Kansas City, MO, USA) to obtain a lyophilised powder form of the extract. The lyophilised extract powder was stored at −20 °C.

The lyophilised powder (equivalent to 0.5 g raw material) was dissolved in 1 mL deionized water and solubilized. Next, the mixture was diluted with methanol to a final volume of 9 mL. The solution was vortexed for 30 s, mixed ultrasonically for 15 min and centrifuged at 10,000 rpm for 10 min. The final supernatant was filtered through a 0.22 μm syringe filter before use.

#### 2.2.2. Instruments and Conditions

Chromatographic fingerprinting analysis of SWHD was performed on an Agilent 1200 series liquid chromatography system (Agilent Technologies, Santa Clara, CA, USA) that consisted of a binary pump (Agilent G1312B), an auto-sampler (Agilent G1329B), and a DAD-vis detector (Agilent G1316A). The data were recorded and analyzed using the Agilent Chemstation software (G1701-97063) for the LC-3D (Liquid Chromatogram-three dimensional) system (Rev. B.04.03-SP1). A Cosmosil 5C18-AR-II column (5 μm, 4.6 mm × 250 mm; Nacalai Tesque Co., Inc., Tokyo, Japan) was used at 25 °C. To detect acteoside, baicalin, wogonoside, and baicalein, the mobile phase was composed of (A) a phosphoric acid aqueous solution (0.4%, *v*/*v*) and (B) acetonitrile, and a gradient elution was performed as follows: 20–45% (B) at 0–15 min and 45% (B) at 15–20 min. The detection wavelength was set at 330 nm. To detect matrine, sophocarpine, oxymatrine, and oxysophocarpine, the mobile phase was composed of (A) a phosphoric acid aqueous solution (0.4%, *v*/*v*) and (B) methanol, and an isocratic elution of 6:94 was used. The detection wavelength was set at 210 nm. The sample injection volume was 10 μL, and the flow rate was 1.0 mL/min.

### 2.3. Virus and Cells

Madin–Darby canine kidney (MDCK) cells (American Type Culture Collection, Manassas, VA, USA) were grown in Dulbecco’s modified Eagle’s medium with 10% foetal bovine serum, 100 U/mL penicillin, and 100 μg/mL streptomycin. The PR8 (Puerto Rico-8) virus strain was obtained from the virus strain collection of the Institute of Virology (Wuhan, China). The virus was propagated and adapted as previously described [15]. The 50% tissue culture infective dose (TCID50) of the virus in the MDCK cells and the 50% lethal dose (LD_50_) of the virus in mice were determined using the Reed–Muench method (TCID_50_ = 10^−7^/100 μL and LD_50_ = 10^−4.5^/50 μL). All cell experiments were infected with the 100 TCID_50_ of the virus. Virus stocks were collected and stored at −80 °C.

### 2.4. Cytotoxicity Assay

The 50% cytotoxic concentration (CC_50_) of the SWHD extract for the MDCK cells was determined using a CCK-8 (Cell Counting Kit-8) assay [16]. The serial dilutions of the SWHD extract for the cytotoxicity assay were 0, 0.98, 1.95, 3.91, 7.81, 15.63, 31.25 and 62.50 mg/mL, and those of oseltamivir were 0, 19.53, 39.06, 78.13, 156.25, 312.50 and 625 μg/mL. The CC_50_ values were calculated using regression analysis.

### 2.5. In Vitro Antiviral Tests

The experiments were performed to investigate the antiviral efficacy of SWHD with different concentrations and the influence of different drug treatment applications on its efficacy. The antiviral activities of the SWHD extract (0.06, 0.12, 0.24, 0.49, 0.98, and 1.95 mg/mL) and oseltamivir (78.13 μg/mL) were determined using confluent cultures of MDCK cells [17]. Briefly, the MDCK cells were treated in several ways. For the antiviral effect assay, 1 × 10^4^ cells /well were plated in 96-well culture plates at 37 °C under 5% CO_2_ for 24 h and inoculated with a mixture of 100 TCID50/well virus and various concentrations of SWHD and oseltamivir in triplicate at 37 °C for 2 h. After supplementation with an overlay medium, they were cultured at 37 °C under 5% CO_2_ for three days. For the penetration assay, the cells were seeded and incubated for 24 h and inoculated with various concentrations of SWHD and oseltamivir at 37 °C for 2 h in triplicate. The medium was then replaced with fresh medium containing 100 TCID50/well virus for another 2 h. After 2 h of infection, the free virus was removed. The cell monolayer was washed with phosphate-buffered saline (PBS) three times and covered with overlay medium. For the attachment assay, the cells were seeded and incubated for 24 h and pre-chilled at 4 °C for 1 h. The medium was replaced with a mixture of 100 TCID50/well virus and various concentrations of SWHD and oseltamivir. After incubation at 4 °C for another 3 h, the free virus was removed. The cell monolayer was washed with ice-cold phosphate-buffered saline (PBS) three times, covered with overlay medium, and incubated at 37 °C under 5% CO_2_ for an another 72 h. For the internalization assay, the cells were seeded, incubated for 24 h and pre-chilled at 4 °C for 1 h. The cells were infected with 100 TCID50/well virus and incubated at 4 °C for another 3 h. The virus-containing medium was replaced with a fresh medium containing various concentrations of SWHD and oseltamivir in triplicate. They were shifted to a culture at 37 °C. At 60 min intervals following the 37 °C shift, the un-internalized virus was inactivated by supplementation with acidic PBS (pH 3) for 1 min, followed with alkaline PBS (pH 11) for neutralization. Then, PBS was replaced with a fresh overlay medium and incubated at 37 °C for an additional 72 h, as previously described [18]. After 72 h of incubation at 37 °C, the cells were fixed with 100 μL of a 10% formaldehyde solution for 1 h. After the solution was removed, the cells were stained with a 0.1% (*w*/*v*) crystal violet solution for 15 min at room temperature. The plates were washed and dried. Optical density (OD) was determined at 570 nm, and the percentage of inhibition was calculated compared with that in virus control.

### 2.6. Time-of-Addition Assay

The antiviral activity of the SWHD extract was examined at different time points prior to and after viral inoculation by crystal violet staining [19]. The cells were seeded and incubated for 24 h as previously described. Various concentrations of SWHD extract were supplemented at 2 h (−2 h), 4 h (−4 h), 8 h (−8 h) or 12 h (−12 h) prior to viral inoculation, or 2 h (+2 h), 4 h (+4 h), 8 h (+8 h) or 12 h (+12 h) after viral inoculation. The supernatants were removed prior to the supplementation of an overlay medium. After 72 h incubation at 37 °C, the monolayer was then fixed with 10% formalin and stained with 1% crystal violet. The percentage of inhibition was calculated as described previously.

### 2.7. Analysis of Viral Gene Amplification by RT-PCR

The effects of SWHD were further evaluated on the expression of the genes encoding haemagglutinin (HA), NA, nucleoprotein (NP), and M2, which are the important proteins of the PR8 virus. Briefly, 4 × 10^5^ cells/well were plated into six-well culture plates for 24 h, and then the cells were infected with 100 TCID_50_ of the virus for 2 h in the presence or absence of the drug. The total intra- and extracellular RNA of the virus was isolated after 48 h of cultivation by using a QIAamp viral RNA mini kit (Qiagen, Germany) according to the manufacturer’s instructions. RT-PCR was performed using the RNA samples, and the conditions were determined following the instruction for SYBR premix Ex Taq II (Tli RNaseH Plus). Amplification was performed using a StepOne real-time PCR system (Mx3005P, Stratagene, La Jolla, CA, USA). The expression of the target mRNA was normalised relative to that of the glyceraldehyde 3-phosphate dehydrogenase (*GAPDH*) gene. Relative quantification was performed based on the 2^−ΔΔ*C*t^ method as previously described [20]. The specific primers for the four target viral genes (namely, NA, HA, M2, and NP) and the housekeeping gene (*GAPDH*) were designed using Primer 5.0 based on the corresponding gene sequences (Table 1).

### 2.8. Indirect Immunofluorescence Assay

The MDCK cells were infected with the PR8 virus. After removing the virus and washing the cells with phosphate-buffered saline (PBS), the cells were incubated with three concentrations of SWHD (0.49, 0.98, and 1.95 mg/mL) diluted in the growth medium. At 48 h post infection, the cells were fixed with 4% paraformaldehyde for 30 min and then permeabilised with 0.1% Triton X-100 for 5 min. After blocking with 2% bovine serum albumin for 20 min, the cells were exposed to a mouse anti-NP monoclonal antibody (1:1000; Zoonogen) at 4 °C for 12 h. The cell nuclei were stained with 4’,6-diamidino-2-phenylindole, and the cells were visualized under a fluorescence microscope [21,22].

### 2.9. Western Blotting

Total protein was extracted from the MDCK cells by using a radioimmunoprecipitation assay buffer (Beyotime, Shanghai, China) and quantified using a bicinchoninic acid assay kit (Bio-Rad, Hercules, CA‎, USA). Equal amounts (30–50 μg) of total protein were loaded and separated using 10% sodium dodecyl sulphate polyacrylamide gel electrophoresis and then transferred to a polyvinylidene difluoride membrane (0.45 μm, Millipore, Billerica, MA, USA). The membranes were blocked in 5% non-fat milk and incubated with primary antibodies overnight at 4 °C. Subsequently, the membranes were incubated with appropriate secondary antibodies for 1 h at 25 °C. Protein bands were developed and quantified by densitometry analysis using an Alpha Innotech imaging system (San Leandro, CA, USA). The results were normalised to those for β-actin.

### 2.10. Mouse Protection and Lung Lesion Assay

Female Balb/c mice weighing 18–22 g were purchased from the Experimental Animal Center, Southern Medical University, China. The animals were housed in groups of six per standard cage, on a 12 h light/dark cycle. The air temperature was maintained at 22 ± 2 °C. This study was approved by the Ethics Committee of Southern Medical University (L2016017, 20160315). The animal research was performed in the ABSL-3 Laboratory of Southern Medical University, and all animals received humane care in compliance with the Chinese Animal Protection Act and the National Research Council Criteria. The resolution number was L2016017. The daily dosage of SWHD extract used on the mice was translated from the clinical dosage for an adult human (60 kg).

To investigate the protective activity of the SWHD extract against PR8 virus in vivo, the mice were anesthetised intraperitoneally with 1% pentobarbital sodium (0.1 mL/20 g) and inoculated intranasally with 50 μL of a viral suspension containing 10 LD50 of the virus (mouse-adapted) or PBS in the normal control group. Two hours later, the inoculated mice then received different concentrations of the SWHD extract (5.85, 11.70, or 23.40 g/kg/day), oseltamivir (0.09 g/kg/day), or PBS daily via gavage for five days. For the mouse protection assay, 10 mice per group were observed for mortality daily for 14 days. The protective effects were estimated by the reduction of mortality and the survival time.

For the lung lesion assay, 10 mice from each group were sacrificed on day five. The lung tissues were harvested and weighed. The lung index was expressed as the ratio of the mean lung weight to the mean body weight. Every lung tissue from each group was divided into two parts, one of them was fixed in 10% phosphate-buffered formalin, and then embedded in paraffin, sectioned, and stained with haematoxylin and eosin (H&E); and the other was ground and centrifuged, and the supernatant was used to test the virus titres in the MDCK cells. Furthermore, the NP and HA proteins of the treated and untreated mice with virus infection were analyzed using Western blot. Finally, the expression of the four target viral genes (NA, HA, M2, and NP) was determined using RT-PCR according to the methods described in Section 2.6.

### 2.11. Data Analysis

Results are expressed as mean ± standard deviation (S.D.). The percentage of control (infection rate; %) was calculated from the plaque counts of the experimental groups divided by those of the virus control. Data were analyzed using analysis of variance (ANOVA) by SPSS ver. 19.0 (Armonk, NY, USA). Tukey’s honest significant difference (HSD) test was used for post-hoc ANOVA comparisons. Statistical significance was considered at *p* < 0.05.

## 3. Results

### 3.1. HPLC Profile of SWHD

**Figure 1:**
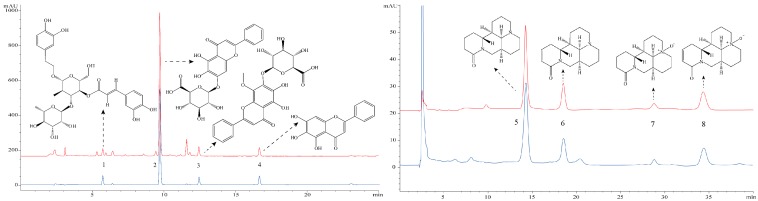
Identification analysis of acteoside (1), baicalin (2), wogonoside (3), baicalein (4), matrine (5), sophocarpine (6), oxymatrine (7), and oxysophocarpine (8) in SWHD by HPLC-DAD in comparison with standard reference compounds. The red colors are represented standard substances and the blue colors are represented samples.

The eight bioactive constituents of SWHD, namely, acteoside, baicalin, wogonoside, baicalein, matrine, sophocarpine, oxymatrine, and oxysophocarpine, were quantified using HPLC in comparison with standard reference compounds (Figure 1). The contents of these compounds in SWHD were 0.95, 82.83, 21.38, 2.11, 8.77, 2.33, 0.63, and 1.91 mg/g, respectively.

### 3.2. Dose- and Time-Dependent Anti-Influenza Activities of SWHD In Vitro

**Figure 2:**
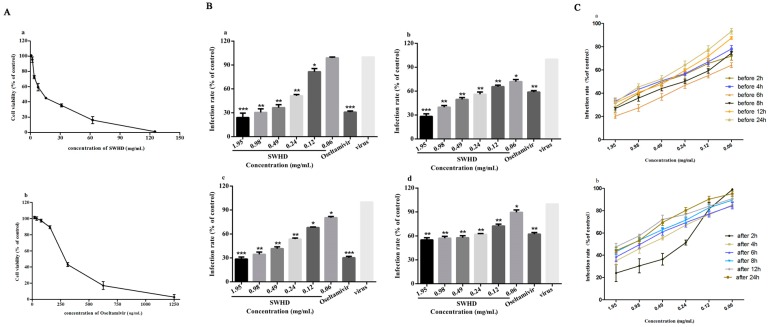
Inhibition the activity of the PR8 virus when given viral inoculation in different stages in vitro. (**A**) The CC50 of the SWHD extract (**a**) and oseltamivir (**b**) for MDCK cells were determined using a CCK-8 assay; (**B**) the inhibitory activity against the virus was determined in vitro by measuring the inhibition of the virus-induced cytopathic effect on cells by crystal violet staining. Antiviral effect assay (**a**); penetration assay (**b**); attachment assay (**c**); internalization assay (**d**); (**C**) the time-dependent inhibitory activity of the SWHD against the virus in vitro: pre-treatment mode (**a**); post-treatment mode (**b**). The values are presented as the means ± S.D. of three individual experiments. * *p* < 0.05; ** *p* < 0.01; *** *p* < 0.001, when compared to the viral control.

To evaluate the antiviral effect of SWHD on the PR8 virus, we tested the ability of SWHD to inhibit the production of the infectious progeny virus in the MDCK cells. The cytotoxicity of SWHD and oseltamivir was tested using the CCK-8 assay. The estimated CC50 values of SWHD and oseltamivir were 12.76 mg/mL and 231.05 μg/mL, respectively (Figure 2A). No cytotoxicity was observed at 78.13 μg/mL oseltamivir and 1.95 mg/mL SWHD. Therefore, the 0.06–1.95 mg/mL concentrations of SWHD and 78.13 μg/mL, the concentration of oseltamivir were selected for the subsequent experiments. The inhibitory activity against the virus was determined in vitro (Figure 2B). Results showed that the viral activities were dramatically reduced, compared with those in the virus control, by SWHD at all the concentrations upon antiviral effect assay (a), penetration assay (b), and attachment assay (c) (*p* < 0.05). At concentrations higher than 0.12 mg/mL, the viral activities were reduced, compared with those in the virus control group, upon internalization assay (d) (*p* < 0.05). Additionally, the viral activities were dramatically reduced by oseltamivir in all treatments (*p* < 0.01). To determine whether the inhibition of the virus replication and the production of an infectious progeny by SWHD were time-dependent, the drug was added immediately after virus inoculation or at indicated times (Figure 2C). Results showed that SWHD treatment caused dose- and time-dependent reductions of viral activity. 

### 3.3. Reduction of Viral Gene Amplification in the Infected MDCK Cells

**Figure 3:**
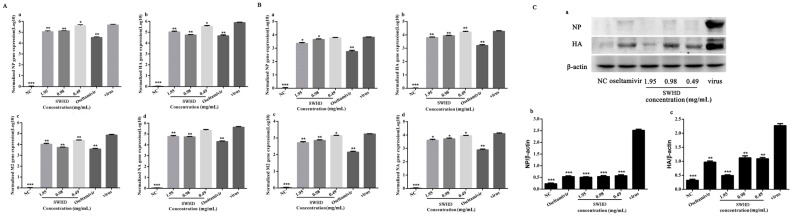
(**A**,**B**) The qPCR results of the quantification of viral mRNA expression of the four genes NP (**a**), HA (**b**), M2 (**c**) and NA (**d**) both in the MDCK cells (**A**) and supernatants (**B**). (**C**) The expression of the NP and HA proteins in the cells were detected by Western blot analysis (**a**). The inhibition effect of the NP (**b**) and HA (**c**) were calculated. The values are presented as the means ± S.D. of three individual experiments. * *p* < 0.05; ** *p* < 0.01; *** *p* < 0.001, when compared to the viral control.

Given that SWHD could inhibit the virus plaque formation at different stages of viral replication in a dose- and time-dependent manner, we presumed that SWHD might affect the number of viral particles. NP and M2 are the main constituent proteins of the virus, whereas HA and NA are antigenic proteins. Thus, we supposed that SWHD could diminish the expression of the four proteins, and the results shown in Figure 3 confirm this assumption. The mRNA expression of the four genes was significantly upregulated in the virus-infected groups compared with that in the normal control group (*p* < 0.001), both in the cells (Figure 3A) and supernatants (Figure 3B). In the treatment groups, the mRNA expression of the four genes was significantly downregulated at all concentrations of SWHD (*p* < 0.05), except the concentration of 0.49 mg/mL, which exerted no influence on the expression of NA mRNA in the cells and NP mRNA in the supernatant compared with the levels in the virus-infected group.

### 3.4. Decreased Expression of the NP and HA Proteins in the Infected MDCK Cells

Given that SWHD can attenuate the susceptibility of cells to external pathogens, we presumed that SWHD might affect the susceptibility of the host cells to the viruses. NP and HA are the two major proteins of the PR8 virus, which may be responsible for the increased susceptibility of the host cells to virus infection. SWHD could reduce the expression of NP and HA at the protein level in a dose-dependent manner in uninfected cells (Figure 3C). This result suggested that the treatment with SWHD could diminish the expression of the two proteins of the virus.

### 3.5. Inhibitory Effects of SWHD on the Nuclear Export of the Viral Ribonucleoprotein (vRNP) in the Infected MDCK Cells

**Figure 4:**
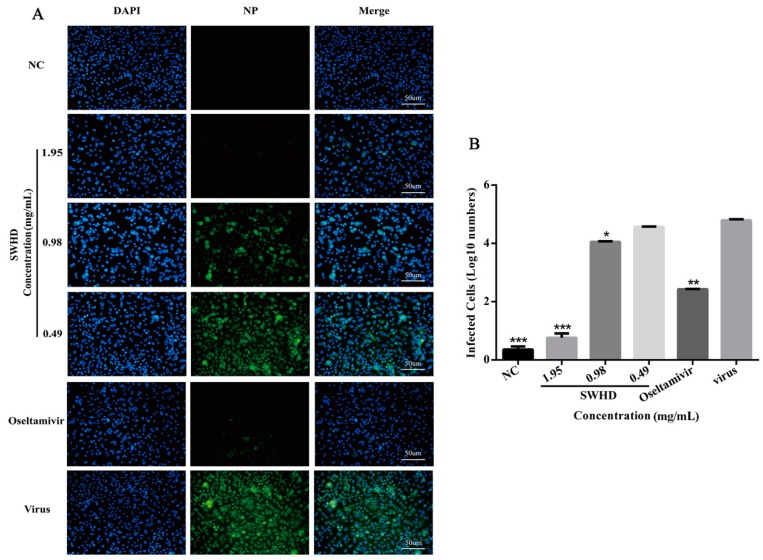
The immunofluorescence assay demonstrated the inhibition of the viral NP protein by SWHD were performed. Cells were then stained for NP (green) and cell nuclei were stained with DAPI (Diphenyl phenylindole) (blue). The scale bar in each panel represents 50 µm (**A**). Images were obtained from one representative experiment of three independent experiments with similar results. The inhibition effect of the NP was calculated at figure (**B**). The values are presented as the means ± S.D. of three individual experiments. * *p* < 0.05; ** *p* < 0.01; *** *p* < 0.001, when compared to the viral control.

Immunostaining assay was performed to assess whether SWHD exerted any effect on the nuclear export of vRNP complexes, an immunostaining assay was performed. NP is a constituent of the vRNP complex, and the nuclear NP retention indicates the inhibition of the vRNP nuclear export. Therefore, we investigated whether SWHD could block the nuclear export of vRNP in the infected MDCK cells. As shown in Figure 4, the immunofluorescence assay demonstrated the inhibition of the viral NP protein by SWHD (0.98 and 1.95 mg/mL) and oseltamivir in the PR8-infected but not in the mock-infected cells. Treatment with SWHD led to a marked reduction in the number of fluorescent cells infected with the virus.

### 3.6. Influence of SWHD on the Survival of the Infected Mice

**Figure 5:**
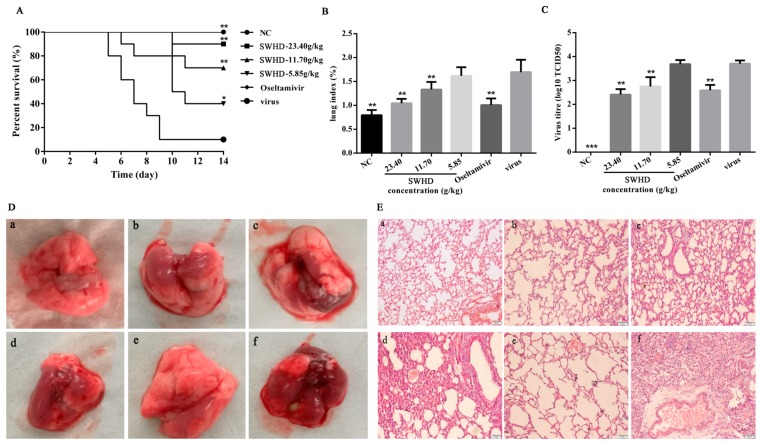
The in vivo protective activities of SWHD extract on lethal IFV infected mice. (**A**) Cum survival curve for indicated groups (*n* = 10 per group). (**B**) Lung index for mice sacrificed at the fifth d.p.i. (*n* = 10 per group). (**C**) Virus titre for mice at the fifth d.p.i. (*n* = 10 per group). (**D**) Morphological changes in lung tissue from mice sacrificed at the fifth d.p.i. (**a**) mock infected mice treated with PBS (normal control, NC); (**b**–**d**) IFV-infected mice treated with 23.40,11.70 or 5.85 g/kg/day of SWHD extract; (**e**) IFV-infected mice treated with oseltamivir; (**f**) IFV-infected mice treated with PBS (viral control). Scale bar = 1 cm. (**E**) Histological observations of lung tissues for mice sacrificed at the fifth d.p.i. Scale bar = 50 µm. (**a**) mock infected mice treated with PBS (normal control, NC); (**b**–**d**) IFV-infected mice treated with 23.40,11.70 or 5.85 g/kg/day of SWHD extract; (**e**) IFV-infected mice treated with oseltamivir; (**f**) IFV-infected mice treated with PBS (viral control). * *p* < 0.05; ** *p* < 0.01, when compared to the viral control.

The experimental mice were monitored for 14 days. Nine mice died after infection in the virus control group, whereas none died in the normal control group (Figure 5A). The mice in the PR8 group presented lack of appetite, inactivity, ruffled fur, a hunched posture, and respiratory distress. Although similar clinical features were observed in the SWHD-treated group, the features were better than those in the virus control group. Furthermore, compared with those in the virus control group, the treatment with SWHD (23.40 g/kg/day) and oseltamivir significantly reduced the mortality, prolonged the lifespan, and increased the survival time of the infected mice (*p* < 0.05).

### 3.7. Effects of SWHD on Influenza Viral Pneumonia

The lung index and inhibition rate of the lung index were calculated to assess the influenza viral pneumonia. As shown in Figure 5B, the lung index was significantly higher in the virus-infected group than in the normal control group (*p* < 0.01). Treatment with SWHD (11.70 and 23.40 g/kg) or oseltamivir significantly decreased the lung index (*p* < 0.01) and increased the inhibition rate of the lung index (Table 2). However, SWHD at 5.85 g/kg exerted no significant effect on the lung index. These results indicate that SWHD (11.70 and 23.40 g/kg) inhibited the virus-induced pneumonia.

### 3.8. SWHD Significantly Decreased the Virus Titres in the Lungs of the Infected Mice

Viral titres reflect the threshold levels of virus needed to initiate infection and the ability to resist viruses rather than the ability to survive. The lung virus titres in the SWHD (11.70 and 23.40 g/kg)-treated and oseltamivir-treated virus-infected mice were all significantly reduced compared with those in the mock-treated infected mice (Figure 5C). The lung virus titre of the mock-treated virus-infected mice was 3.71 ± 0.13. However, the PR8 titres in the lungs of the mice treated with SWHD (11.70 and 23.40 g/kg) or oseltamivir were 2.41 ± 0.23, 2.75 ± 0.38 and 2.59 ± 0.23, respectively, i.e., much lower than those of the mock-treated virus-infected mice. However, the treatment with SWHD at 5.85 g/kg resulted in a titre of 3.69 ± 0.17, i.e., similar to that in the virus control group. This result was consistent with the results of histological changes in the lungs.

### 3.9. Effects of SWHD Treatment on the PR8-Induced Lung Pathological Changes

Morphological changes in the lung tissue of the mice induced and not induced with the PR8 virus infection are shown in Figure 5D. The lung tissue from the control mice was pink and without any congestion. However, the virus-infected mice exhibited a large area of congestion in the lung tissue. The virus-induced lung inflammation was significantly reduced by the treatment with SWHD (5.85, 11.70, and 23.40 g/kg) or oseltamivir. The pathological changes in the lungs of the treated and untreated mice with virus infection are shown in Figure 5E. The mice in the control group showed no obvious histological changes (Figure 5E(a)). The lungs of the virus-infected group showed the presence of necrotic bronchial and bronchiolar epithelia, haemorrhage, alveolar thickening, and marked infiltration of inflammatory cells (Figure 5E(b)). Compared with that in the virus control group, the virus-induced lung inflammation was significantly reduced upon treatment with SWHD (11.70 and 23.40 g/kg) or oseltamivir (Figure 5E(c–e)). However, the treatment with SWHD at 5.85 g/kg resulted in histological changes similar to those in the virus control group (Figure 5E(f)). These results indicated that the treatment with SWHD (11.70 and 23.40 g/kg) could ameliorate the lung damage in the virus-infected mice.

### 3.10. Effects of SWHD on the mRNA and Protein Expression of NP, NA, HA, and M2 in the Lungs of the Infected Mice

**Figure 6:**
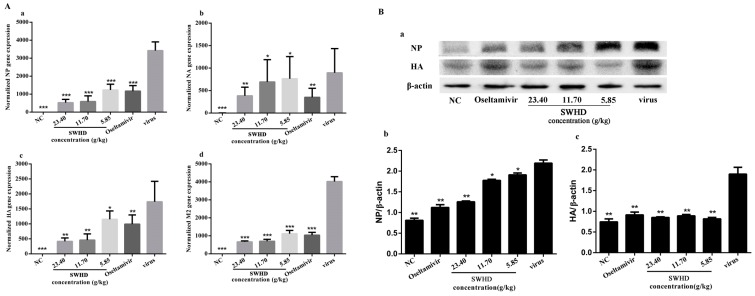
(**A**) The quantification of viral mRNA expression of the four genes NP, M2, HA and NA in vivo by the SWHD extract on lethally IFV-infected mice. (**a**) the mRNA expression of the NP; (**b**) the mRNA expression of the NA; (**c**) the mRNA expression of the HA; (**d**) the mRNA expression of the M2. (**B**) The expression of the NP and HA proteins of the lungs were detected by Western blot analysis. All concentrations of the SWHD and oseltamivir could significantly inhibit the expression of the two proteins compared to the viral control. The values are presented as the means ± S.D. of three individual experiments. * *p* < 0.05; ** *p* < 0.01; *** *p* < 0.001, when compared to the viral control.

Given that the in vitro results showed that SWHD could suppress the expression of the four target virus proteins, we inferred that the mRNA expressions of NP, NA, HA, and M2 and the protein expression of NP and HA might be downregulated in lung tissues of mice by SWHD treatment. Thus, the mRNA and protein expression levels of NP, NA, HA, and M2 were examined in the lungs of the infected mice. As shown in Figure 6, the gene and protein levels significantly increased in the virus group (*p* < 0.05), whereas all of the concentrations of the SWHD administration (5.85, 11.70, and 23.40 g/kg) significantly decreased the expression of the four genes and proteins (*p* < 0.05). This result indicated that SWHD could inhibit the virus replication and proliferation by suppressing the expression of the target virus proteins.

## 4. Discussion

Given the infections caused by the IFV and the adverse reactions and resistance to antiviral drugs, finding new drugs to cure IFV infection is very essential. The purpose of an anti-influenza strategy is to reduce the viral load and reduce the damage of the virus on the respiratory system. Currently, the use of herbal drugs is a popular therapy, and this tendency will continue in the future. An effective drug is not only a bioactive compound, but also an effective component (well-characterized mixtures or extracts). Traditional Chinese medicine may be a potential alternative drug source for drug design and discovery. SWHD is a TCM remedy used to treat influenza and its secondary infections. This study was the first time to demonstrate that SWHD can inhibit PR8 virus infection broadly in vitro and against virus in BALB/c mice in our study.

SWHD could effectively inhibit the PR8 virus at different stages of viral replication; this finding was confirmed by measuring the inhibition of the virus-induced cytopathic effect on the cells by crystal violet staining. Results showed SWHD at concentrations higher than 0.12 mg/mL could dramatically reduce the viral activities compared with those in the virus control, irrespective of the way of administration. Also, the anti-viral effect of high-doses of the SWHD were similar to that of oseltamivir. Pretreatment with SWHD could more dramatically inhibit the viral activities than oseltamivir. This finding indicated that SWHD was more potent in preventing the attachment and penetration of the virus than in directly inhibiting the viral infection. SWHD is a Chinese herbal compound prescription; it is composed of a variety of ingredients that could prevent diseases. SWHD exhibits a broad spectrum of antiviral effects and also regulates immunity, increases the body’s resistance to disease, inhibits viruses at different stages and targets several viral infections. In the current study, SWHD was added either immediately after the virus inoculation or at predetermined times. Results showed that SWHD treatment caused dose- and time-dependent reductions of viral replication. After prolonged administration as the prophylaxis, the antiviral effect of SWHD was gradually enhanced, and the antiviral ability was better than that of oseltamivir. This finding indicated that SWHD showed a significant preventive and therapeutic effect and that SWHD was a safe and effective antiviral drug for treatment of IFV infections combined with the cytotoxic results.

HA, NA, NP, and M2 are the four important proteins that play key roles in the production, replication, proliferation and release of the virus from host cells. After viral transcription and replication, the four proteins are involved in the virus release and attachment to new host cells, which become infected. The popular anti-influenza drugs such as oseltamivir, zanamivir, and amantadine are known NA and M2 blockers. Therefore, these proteins are potential targets for novel antivirals. This study showed, using RT-PCR, that SWHD demonstrated a significant intra- and extracellular anti-IFV activity in the MDCK cell cultures. Results indicated that SWHD could strongly inhibit HA, NA, NP, and the M2 ion channel before the virus attached to the host cells. Furthermore, the expression of the HA and NP proteins was analyzed using Western blot. All concentrations of SWHD used in this experiment, as well as oseltamivir, could significantly inhibit the increase in the number of virus particles, and the anti-influenza activity of SWHD at a concentration of 1.95 mg/mL was higher than that of oseltamivir. The effect of SWHD on the NP protein was further verified using an indirect immunofluorescence assay. Results indicated that the most significant role of viral inhibition by SWHD was blocking the proliferation and replication of the viral particles in vitro and some doses of the SWHD had a better anti-virus effect than oseltamivir.

Some drugs have achieved satisfactory results in vitro. However, they showed poor efficacy and were even completely ineffective when administered to animals. The effect of SWHD has been observed in empirical studies or case reports. Thus, we further verified the antiviral activity of SWHD in mice infected with the PR8 virus. Results indicated that SWHD (11.70 and 23.40 g/kg) significantly reduced the mortality and prolonged the lifespan of the infected mice. The clinical signs of infection were alleviated in the SWHD-treated group. To evaluate the severity of pneumonia, the lung index, virus titres in the lung, and pathological changes in lung tissues were investigated in our study. Lung index is an indicator of the severity of pneumonia and evidently increases in the early phase of IFV infection [23,24]. This paper found that remarkable decreases in the lung indexes and significant reductions of the virus titres in the SWHD (11.70 and 23.40 g/kg) groups. In addition, an excessive immune response, associated with pathological changes in lung tissue, is believed to be a predictor of influenza-mediated death. The pathological analysis of the lung tissues demonstrated evident necrotic bronchial walls, alveolar thickening, and marked infiltration of inflammatory cells in the PR8-infected group. This finding was consistent with the results of previous studies. When the infected mice were treated with SWHD (11.70 and 23.40 g/kg), the pathological damage in the lung tissue was significantly relieved. In addition, the expression levels of NA, HA, NP and M2 mRNA were significantly downregulated in the lung tissues at all doses of SWHD after infection in comparison with the IFV-infected group. The expression levels of the HA and NP proteins were significantly reduced in the lung tissues in the SWHD-treated group in comparison with those in the PR8-infected group. These results indicated that SWHD is an effective oral anti-influenza agent via alleviating lung lesions and reducing viral loads in the lungs and the expression of the target virus proteins.

SWHD is a famous herbal remedy that has been used for thousands of years. In this paper, we characterized the presence of the major ingredients of SWHD (Figure 1). Baicalin, wogonoside, matrine and oxymatrine, etc., exhibit significant antiviral activity against IFVs, dengue virus, enterovirus-71, and Japanese encephalitis virus [25,26,27,28]. The pharmacological activities of these ingredients, including anti-inflammatory, anticancer, cardioprotective, neuroprotective, antibacterial, have also demonstrated. Thus, we hypothesized that baicalin, wogonoside, baicalein, matrine and oxymatrine may be the active components of SWHD against IFV.

In summary, this study demonstrated that SWHD contained broad clear anti-influenza activity in vitro and vivo. The anti-influenza effect was attributed to the blocking of the proliferation and replication of the viral particles. The protective effect on the virus-infected mice was achieved by alleviation of the lung injury and reduction of the lung viral titres and the expression of the virus target proteins. Furthermore, the three SWHD drugs are inexpensive and have a good volume of production. These findings provide a new option for the treatment of IFV infection and information to further reveal the mechanisms of SWHD.

## Figures and Tables

**Table 1:** Primer sequence for qRT-PCR.

Target Gene	Direction	Sequence (5′-3′)
*NP*	Fwd	TGCTTCAAAACAGCCAAGTG
	Rev	GATGCCCTCTGTTGATTGGT
*NA*	Fwd	ACATCTGCAGTGGGGTTTTC
	Rev	ACCAATCAGTCATTGCCACA
*M2*	Fwd	GGGAAGAACACCGATCTTGA
	Rev	GCAAGTGCACCAGCAGAATA
*HA*	Fwd	GCTGCAGATGCAGACACAAT
	Rev	CCCTCAGCTCCTCATAGTCG

**Table 2:** Detection of effect of SWHD on the lung index of infected mice.

Group	Dose (g/kg)	Lung Index (%)	Inhibition Rate of Lung Index (%)
NC	-	0.7946 ± 0.1095 **	-
Virus	-	1.698 ± 0.2572	-
Oseltamivir	0.078	1.007 ± 0.1386 **	40.70
SWHD	23.40	1.045 ± 0.09131 **	38.42
	11.70	1.331 ± 0.1584 **	21.58
	5.85	1.617 ± 0.1809	4.74

NC: normal control group; Virus: virus-infected group; Compared to virus, ** *p* < 0.01.
